# Risk Factors for Urinary Tract Infections in Children with Hematuria in the Emergency Department

**DOI:** 10.3390/children11020248

**Published:** 2024-02-15

**Authors:** Bei-Cyuan Guo, Chun-Yu Chen, Wun-Yan Huang, Wen-Ya Lin, Ying-Ju Chen, Tai-An Lee, Mao-Jen Lin, Han-Ping Wu

**Affiliations:** 1 Department of Pediatrics, National Cheng Kung University Hospital, College of Medicine, National Cheng Kung University, Tainan 70403, Taiwan; n109676@mail.hosp.ncku.edu.tw; 2 Department of Emergency Medicine, Tungs’ Taichung MetroHarbor Hospital, Taichung 43503, Taiwan; t14367@ms3.sltung.com.tw; 3 Department of Pediatric Emergency Medicine, China Medical University Children’s Hospital, Taichung 40447, Taiwan; d33901@mail.cmuh.org.tw; 4 Department of Pediatrics, Taichung Veteran General Hospital, Taichung 43503, Taiwan; wenya@vghtc.gov.tw; 5 Department of Rehabilitation, New Tai Ping Cheng Ching Hospital, Taichung 41142, Taiwan; ortho19486@gmail.com; 6 Department of Emergency Medicine, Chang Bing Show Chwan Memorial Hospital, Changhua 50544, Taiwan; diane.lee.em@gmail.com; 7 Division of Cardiology, Department of Medicine, Taichung Tzu Chi Hospital, The Buddhist Tzu Chi Medical Foundation, Taichung 42743, Taiwan; 8 Department of Medicine, College of Medicine, Tzu Chi University, Hualien 97002, Taiwan; 9 College of Medicine, Chang Gung University, Taoyuan 33302, Taiwan; 10 Department of Pediatrics, Chiayi Chang Gung Memorial Hospital, Chiayi 61363, Taiwan

**Keywords:** hematuria, urinary tract infection, pediatric emergency department, non-pyuria, predictor

## Abstract

Introduction: Hematuria is a worrisome symptom in children and is sometimes associated with urinary tract infections (UTIs). This study aimed to identify useful clinical factors that can predict UTIs in hematuria patients without pyuria in the pediatric emergency department (ED). Methods: We retrospectively recruited patients with hematuria from the pediatric ED. Clinical symptoms, urine biochemistry and microscopic examination results, and blood laboratory tests were analyzed to identify the predictors of UTIs. Patients were divided into the verbal group (age ≥ 2 years) and non-verbal group (age < 2 years) for identifying predictors of UTIs. Causes of hematuria were also investigated. Results: A total of 161 patients with hematuria without pyuria were evaluated. Among symptoms, dysuria was significantly correlated with UTIs. Regarding urine biochemistry data, urine esterase and urine protein > 30 mg/dl were found to be significant parameters for predicting UTIs, while urine esterase and urine nitrite showed significant differences in children with age < 2 years. In the urine microscopic examinations, urine red blood cells (RBC) > 373/µL in children aged ≥ 2 years and urine RBC > 8/µL in children aged < 2 years were associated with UTIs. In addition, UTIs and urinary tract stones were found to be the top two causes of hematuria. Conclusions: Dysuria, urine esterase, urine nitrite, and urine protein may be useful parameters for predicting UTIs in pediatric patients with hematuria but no pyuria in the ED. In addition, a UTI was the most commonly identified etiology of hematuria without pyuria, followed by urinary tract stones.

## 1. Introduction

Urinary tract infections (UTIs) are the most common bacterial infection in children. The clinical presentation of UTIs in children is highly heterogeneous and the diagnosis may not be straightforward. In clinical practice, children with a suspected UTI in pediatric emergency departments (EDs) can be diagnosed early by urinalysis and confirmed by urine culture. In urinalysis, pyuria has a specificity of approximately 81% and sensitivity of 73% for UTIs [1]. However, sometimes, pyuria may not be present and only hematuria is noted in children or adults with a UTI [2,3].

In some studies, the prevalence of asymptomatic hematuria detected by urinalyses in school-aged children was about 4% [4]. Hematuria may be divided into macroscopic and microscopic hematuria. Macroscopic hematuria is defined as the presence of an increased number of red blood cells (RBCs) in the urine that are visible to the naked eye, resulting in a reddish discoloration of the urine [5]. Microscopic hematuria is diagnosed by the presence of over five RBCs/µL in the urine specimen [5,6]. Hematuria is also classified into persistent hematuria or transient hematuria. Persistent hematuria is defined as more than 4 to 6 weeks of positive urine examination results. Transient hematuria has been found to occur in association with fever, exercise, UTIs, and trauma [7].

In patients with hematuria without pyuria accompanied by fever or lower urinary tract symptoms, it is difficult to rule out a UTI. The present study aimed to investigate factors that can help predict UTIs in patients with hematuria in the pediatric ED.

## 2. Materials and methods

### 2.1. Patient Population

This study was a chart review of pediatric patients with hematuria aged 18 years or younger who were admitted to a pediatric ED in a medical center in central Taiwan from 2012 to 2022. Urine data were collected from pediatric patients who underwent urine examination, retrieved from electronic medical records. The urine was analyzed using iQ200 Series Automated Urine Microscopy Analyzers in the Department of Laboratory Medicine. The data on hematuric patients in the pediatric ED were collected. All pediatric patients with isolated hematuria and no pyuria based on urine examination were included. In addition, we excluded patients who were using antibiotics before visiting the pediatric ED, those with underlying diseases causing hematuria, recent trauma history, congenital genitourinary anomalies, female patients with menstruation, and those who had ingested food or drugs known to cause red or pink discoloration of urine. The patients were divided into four age groups: infant and toddler (<2 years), preschool age (2 to <7 years), school age (7–13 years), and adolescent (13–18 years). This study was approved by the Institutional Review Board and Ethics Committee of China Medical University Hospital (No: CMUH109-REC3-087). All procedures were conducted in accordance with clinical necessities. Data were collected, reviewed, de-identified, and anonymized before analysis, and the Ethics Committee waived the requirement for informed consent because of the anonymized nature of the data and scientific purpose of the study.

### 2.2. Study Design

All included patients were divided into two groups based on the causes of hematuria (urinary tract infection and other causes). Hematuria was defined as the presence of over 5 RBCs/µL in the urine specimen [5,6]. Pyuria was defined by the threshold of 25 white blood cells (WBC)/µL in urine examination [8]. The criteria for diagnosing UTIs in children include any number of colony-forming units (CFU) per milliliter of urine specimen from suprapubic bladder puncture. Clean-catch urine, midstream, and catheterization urine cultures can be considered positive at 10^3^–10^4^ cfu/mL of a monoculture [9,10,11]. Clinical data including age, sex, triage with vital signs in the ED, previous history of hematuria, family history, underlying diseases, and previous treatment were collected and analyzed. All data from baseline clinical assessment, physical examination, clinical symptoms such as gastrointestinal, UTI, or other symptoms, urine examination, blood laboratory tests, and common imaging examination (including radiography or computed tomography) were also recorded. The findings of urine examination (biochemistry and microscopic examination) and urine culture were collected in detail. In addition, the findings of special laboratory examinations such as C3, C4, antinuclear antibody (ANA), and antistreptolysin O (ASLO), and imaging investigations, including renal ultrasonography, Tc-99m-DMSA renal cortical scan, and intravenous pyelography performed during hospitalization or in out-patient departments during follow-up, were recorded.

The correlation of the variables with UTIs and other causes was further analyzed and compared across the different age groups: non-verbal children (<2 years) and verbal children (2–18 years). All clinical factors and laboratory findings were analyzed to identify the parameters for predicting UTIs in children with hematuria without pyuria in urinalysis. Moreover, in non-UTI children with hematuria, the possible etiologies of hematuria were also analyzed based on the medical records.

### 2.3. Statistical Analysis

Differences between groups and predictive values were calculated using ANOVA, the chi-squared test, the Mann–Whitney U test, and Yates’ continuity correction for statistical analysis. In the descriptive analysis, values were presented as means ± standard deviation (SD). The differences between the groups were presented as 95% confidence intervals. Statistical significance was defined at *p* < 0.05, and all statistical analyses were conducted using IBM SPSS Statistics software (version 22.0; SPSS Inc., Chicago, IL, USA).

## 3. Results

### 3.1. Demographics

**Figure 1:**
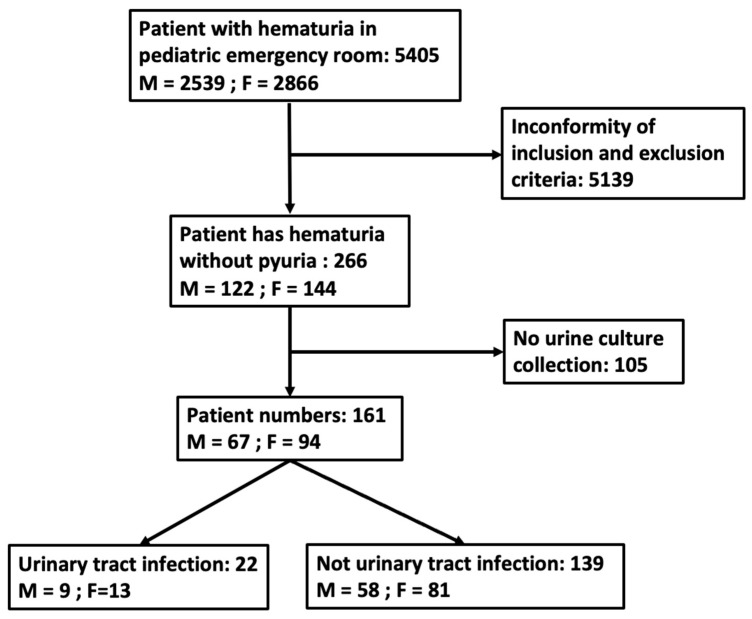
A flow chart quantifying the number of hematuria patients without pyuria.

**Figure 2:**
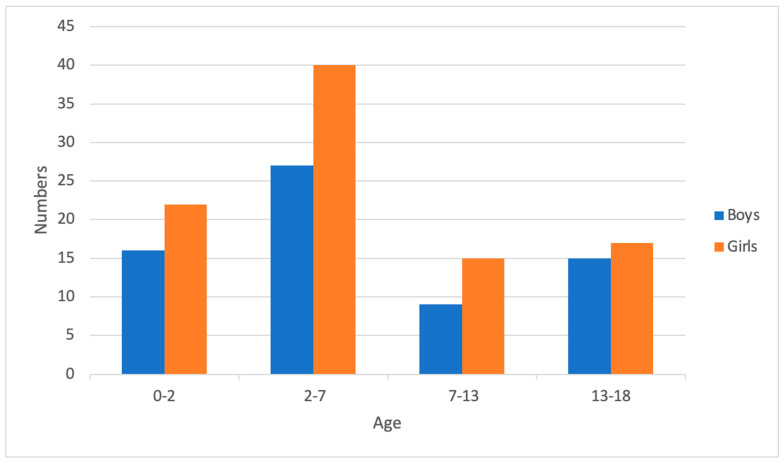
Distribution of all 161 hematuria patients without pyuria among the different age groups.

A total of 5405 patients presented with hematuria in the pediatric ED. After excluding cases with both hematuria and pyuria, 266 patients were included; subsequently, 105 patients were excluded because of no collection of urine culture. Eventually, 161 patients were enrolled in the study (Figure 1). The patients were discharged or admitted for further treatment based on their clinical condition. The mean age was 6.0 years. Female patients were predominant, with a female/male ratio of 1.40. About one fourth of the patients were below 2 years of age (n = 38, 23.6%). In addition, 83 patients had fever; other common symptoms and signs were gross hematuria (33.5%) and dysuria (24.8%) (Table 1). The distribution of the 161 hematuria patients into UTI and non-UTI groups is shown in Figure 2. Fever was the most common symptom in the younger age groups and the proportion decreased with age. Gross hematuria, lower abdominal pain, flank pain, and dysuria occurred frequently in verbal children. Among blood tests, lower C-reactive protein (CRP) levels and higher urine RBC concentration were found in the older age groups (Table 2).

### 3.2. Causes of Hematuria and UTI Pathogen

A UTI was the most common cause identified in children with only hematuria in the ED (N = 22, 13.7%), followed by urinary tract stones (N = 8) and nephrocalcinosis (N = 7) in our study. The other causes included hypercalciuria (N = 6), hydronephrosis (N = 3), glomerulonephritis (N = 1), Wilms tumor (N = 1), and renal angiomyolipoma (N = 1). However, the cause of hematuria was not identified in most of the cases (N = 112, 69.6%). The top two infectious pathogens responsible for UTIs in our study were *Escherichia coli* (N = 12) and *Enterococcus faecalis* (N = 3). The other pathogens included *Enterobacter aerogenes, Klebsiella pneumoniae, Proteus mirabilis, Staphylococcus saprophyticus, Citrobacter koseri, Yeast,* and GPB, each counted as 1.

### 3.3. Presentation of Hematuria in Pediatric ED Patients without Pyuria

The results of the comparison of clinical factors between the UTI and non-UTI groups are shown in Table 3. Among these factors, dysuria, urine esterase, urine protein over 30 mg/dL, and blood WBCs over 9300/mm^3^ were statistically significant in the UTI group. UTIs were more common in children with hematuria who were aged between 13 and 18 years compared to other age groups (*p* < 0.05)

### 3.4. Prediction of UTIs in Pediatric Patients with Hematuria Based on the Two Age Groups

Patients were divided into verbal and non-verbal groups for UTI prediction. In patients aged greater than 2 years, dysuria was a significant factor for predicting UTIs. In urinalysis, urine RBCs over 373/µL and urine protein over 30 mg/dL were significant factors for predicting UTIs (Table 4). However, in patients less than 2 years old, although fever was found in all cases with UTIs (100%), no statistically significant difference was noted. In urinalysis, urine nitrite, urine esterase, and urine RBC > 8/µL showed significant differences for predicting UTIs (Table 5).

## 4. Discussion

A UTI leads to a localized inflammatory reaction that can cause pyuria and the presence of leukocyte esterase in the urine [4]; however, sometimes pyuria may not be noted in children with UTIs. Until now, no previous study has fully explored the correlation between hematuria without pyuria and UTIs. In the present study, many useful factors were identified to predict UTIs in children with only hematuria admitted to the pediatric ED.

Varied considerations for UTIs are observed across different age groups in children.

A detailed medical history and a complete physical examination are important for the diagnosis of UTIs. Specifically, in acutely ill children less than 3 months old, urinalysis is recommended irrespective of symptoms [12,13]. In the age range of 3 months to 24 months, urinalysis can be performed in the presence of fever or based on identified risk factors such as being an uncircumcised boy, having a history of UTIs, experiencing pain or crying during urination, or having malodorous urine [14,15]. In children aged over 2 years and verbal children with the presence of urinary symptoms (dysuria, urinary frequency, hematuria, abdominal pain, back pain, or new daytime incontinence) and younger children with inexplicable fever, UTIs should be considered, and urine examination and urine culture must be performed [1,14,15]. We not only compared pediatric patients with hematuria without pyuria based on diagnosis but also distinguished them through subgroups by age (<2 and ≥2 years). In our study, we found that only dysuria was a significant factor for predicting UTIs in children with hematuria without pyuria.

Clinically, urine examination is an easy and quick method for identifying children with suspected UTIs in the ED. Many parameters of urine examination may be helpful for predicting UTIs, including leukocyte esterase, urine nitrite, urine occult blood, and proteinuria, but the true predictive power of these parameters for diagnosing UTIs may be variable [16,17,18,19,20,21,22]. Urine culture demonstrates a high specificity for predicting UTIs, but the results are not available immediately. In our study, urine biochemistry for urine esterase, urine nitrite, and urine protein over 30 mg/dl were found to be useful parameters for diagnosing UTIs in children with hematuria. Among the blood tests, WBC, CRP, and procalcitonin (PCT) are broadly used for the evaluation of bacterial infection [23]. In the present study, WBC count and CRP level showed no significant difference between the UTI group and non-UTI group.

In our study, UTIs (13.7%) and urinary tract stones (5.0%) were the two most common etiologies of hematuria without pyuria in children. The causes of hematuria in children are variable and include glomerular diseases, interstitial and tubular diseases, hematologic causes, urinary tract diseases, structural anomalies, medications, and no identifiable diagnosis [24,25,26]. An acute bacterial urinary tract infection is the most common cause of gross hematuria in children [27]. Meanwhile, the most common causes of microscopic hematuria include benign familial hematuria, hypercalciuria, and immunoglobulin A (IgA) nephropathy [28]. In cases of recurrent gross hematuria in children, consideration should be given to IgA nephropathy, Alport syndrome, or thin glomerular basement membrane disease [29]. For persistent microscopic hematuria in children, although the majority of cases are idiopathic, the most common causes include hypercalciuria (with or without kidney stones), urinary tract infections, urinary tract malformations (including nutcracker syndrome), disorders of the glomerular basement membrane (Alport syndrome), and IgA nephropathy [30]. If gross hematuria recurs after the third episode, or if it is associated with hypertension or renal function impairment, or if microscopic hematuria is associated with the detection of dysmorphic red blood cells in the Addis count test (≥70% increase) or significant proteinuria, renal biopsy is indicated [31]. After 2020, the COVID-19 pandemic caused severe damage in children worldwide [32,33,34,35], even leading to multisystem inflammatory syndrome (MIS-C) in children [36,37]. Hematuria might also be a sign of MIS-C and should be listed in the differential diagnosis for hematuria [38]. A review article provided an algorithm for evaluating hematuria in children [39]. Clinicians need to carefully consider medical history, perform a physical examination, and then use the flow chart to find the cause of hematuria. However, in our study period, not all hematuria patients were assessed by the flow diagram, and many patients discharged from the pediatric ED did not return to the outpatient department for reappraisal of hematuria. This may be the reason for no cause being identified in most of the hematuria cases (69.6%) in our study.

### 4.1. Limitations

The present study has some limitations. First, since this was a retrospective single-center review of medical records, some details of the clinical presentation may not be stringently recorded. Second, not all patients with non-UTI hematuria had a definite diagnosis because many patients were lost in the follow-up; therefore, the causes of hematuria could not be investigated in some patients.

### 4.2. Future prospect

In this study, the causes of a significant number of pediatric patients with hematuria remain unidentified, highlighting the imperative to establish a clinical flow chart for hematuria diagnosis and treatment to decrease the percentage of loss follow-up or non-diagnosis. On the other hand, the definitions of hematuria and pyuria vary across studies [5,6,8,40,41], and in pediatric patients with hematuria but without pyuria, approximately 40% of cases (N = 105) were excluded due to a lack of urine culture collection. Further research can be conducted by employing different definitions for pyuria and hematuria and collecting urine culture in these patients to expand and refine the results.

## 5. Conclusions

Dysuria, urine esterase, urine nitrite, and urine protein may be useful parameters for predicting UTIs in pediatric patients with hematuria without pyuria in the ED. In addition, UTIs were the most commonly identified etiology of non-pyuria hematuria, followed by urinary tract stones.

## Figures and Tables

**Table 1:** Demographics.

Items	N (%/Mean ± SD)
Numbers	161
Mean age (years)	6.0 ± 5.5
Gender (M/F)	67: 94
Clinical presentation	
Fever (%)	83 (51.6)
Fever (highest temperature at PER, °C)	39.3 ± 0.8
Gross hematuria	54 (33.5)
Cough	26 (16.1)
Rhinorrhea	26 (16.1)
Nausea or vomiting	17 (10.6)
Diarrhea	3 (1.9)
Upper or periumbilical pain	5 (3.1)
Lower abdominal pain	10 (6.2)
Flank pain	17 (10.6)
Dysuria	40 (24.8)
Urine frequency	10 (6.2)
Urine discharge	2 (1.2)
Laboratory data examinations	
WBC (/mm^3^)	89 (55.2/10,991 ± 5364)
Neutrophil (%)	89 (55.2/59.7 ± 16.4)
CRP (mg/dl)	85 (52.8/3.46 ± 5.69)
Urine RBC (/µL)	161 (100/271 ± 410)
Urine WBC (/µL)	161 (100/10 ± 8)
Hospitalization (days)	44 (27.3/4.8 ± 3.7)
Antibiotics treatment	90(55.9)

WBC: white blood cell; RBC: red blood cell; CRP: C-reactive protein.

**Table 2:** Demographics, distinguished by age.

Items	N (%/mean ± SD)				*p*-Value
Ages (Years)	Age < 2	2 ≤ Age < 7	7 ≤ Age < 13	13 ≤ Age < 18	
Mean age (years) ^a^	0.7 ± 0.5	3.5 ± 1.2	8.5 ± 1.4	15.6 ± 1.3	<0.001
Numbers	38	67	24	32	
Gender (M/F)	16/22	27/40	9/15	15/17	0.908
Clinical presentation					
Fever ^a^	36 (94.7)	35 (52.2)	7 (29.1)	5 (15.6)	<0.001
Fever (highest temperature at PER, °C)	39.3 ± 0.9	39.4 ± 0.7	39.3 ± 0.6	38.6 ± 0.5	0.068
Gross hematuria ^a^	3 (7.9)	22 (32.8)	10 (41.7)	19 (59.4)	<0.001
Cough	6 (15.8)	12 (18.0)	6 (25.0)	2 (6.3)	0.255
Rhinorrhea	6 (15.8)	11 (16.4)	7 (29.2)	2 (6.3)	0.156
Nausea or vomiting	4 (10.5)	8 (12.0)	2 (8.3)	3 (9.4)	1.000
Diarrhea	2 (5.2)	1 (1.5)	0 (0)	0 (0)	0.341
Upper or periumbilical pain	0 (0)	3 (4.5)	1 (4.2)	1 (3.1)	0.651
Lower abdominal pain ^a^	0 (0)	0 (0)	1 (4.2)	9 (28.1)	<0.001
Flank pain ^a^	0 (0)	2 (3.0)	2 (8.3)	13 (31.3)	<0.001
Dysuria ^a^	1 (2.6)	21 (31.3)	9 (37.5)	9 (28.1)	<0.001
Urine frequency	0 (0)	4 (6.0)	1 (4.2)	5 (15.6)	0.056
Urine discharge	1 (2.6)	1 (1.5)	0 (0)	0 (0)	1.000
Laboratory data examinations					
WBC (/mm^3^)	11,445 ± 7670	11,762 ± 5259	9503 ± 2510	10,337 ± 3376	0.300
Neutrophil (%) ^a^	49.4 ± 18.7	60.6 ± 14.2	65.3 ± 14.3	66.2 ± 12.2	0.007
CRP (mg/dL) ^a^	4.44 ± 8.34	4.26 ± 4.92	2.13 ± 2.70	1.39 ± 2.25	0.018
Urine RBC (/µL) ^a^	44 ± 160	225 ± 372	300 ± 395	642 ± 460	<0.001
Urine WBC (/µL)	12 ± 8	10 ± 7	8 ± 7	10 ± 8	0.158
Hospitalization (numbers) ^a^	18	20	3	3	0.001
Hospitalization (days) ^a^	4.4 ± 1.9	5.2 ± 5.1	6.3 ± 3.2	2.7 ± 0.6	0.048
Antibiotics treatment ^a^	14 (36.8)	40 (59.7)	17 (70.8)	19 (59.4)	0.040

WBC: white blood cell; RBC: red blood cell; CRP: C-reactive protein; ^a^ *p*-value < 0.05.

**Table 3:** The correlation of variables with different diagnoses causing hematuria without pyuria (N = 161).

Variables		UTI (n = 22)		Other Causes, non-UTI (n = 139)		*p*-Value
		N	%	N	%	
Age						
0–2		5	22.7	33	27.4	1.000
2–7		7	31.8	60	43.2	0.316
7–13		1	4.6	23	16.6	0.252
13–18 ^a^		9	40.9	23	16.6	0.008
Male sex		9	40.9	58	41.7	0.942
Clinical symptoms						
Fever		9	40.9	74	53.2	0.282
Gross hematuria		8	36.4	46	33.1	0.746
Cough		1	4.6	25	18.0	0.201
Rhinorrhea		1	4.6	25	18.0	0.201
Nausea or vomiting		2	9.1	15	10.8	1.000
Diarrhea		0	0	3	2.2	1.000
Urine discharge		0	0	2	1.4	1.000
Urine frequency		3	13.6	7	5.0	0.281
Dysuria ^a^		11	50	29	20.9	0.003
Upper or periumbilical pain		0	0	5	3.6	0.808
Lower abdominal pain		3	13.6	7	5.0	0.281
Flank pain		4	18.2	13	9.4	0.380
Hospitalization		7	31.8	37	26.6	0.611
Urine examination						
Urine OB	+	17	77.3	79	56.2	0.114
Urine protein	+	13	59.1	73	52.5	0.566
Urine nitrite	+	2	9.1	2	1.4	0.160
Urine esterase ^a^	+	5	22.7	5	3.6	0.003
Urine bacteria	+	1	4.6	4	2.9	1.000
Cutoff values						
Age > 8 years ^a^		10	45.5	32	23.0	0.026
Urine protein > 30 mg/dL ^a^		7	31.8	13	9.4	0.003
Blood WBC amount > 9300/mm^3 a^		13	86.7	40	53.3	0.042
Variables’ population means		Mean ± SD (Number)		Mean ± SD (Number)		
Age (years) ^a^		8.1 ± 6.4 (22)		5.6 ± 5.3 (139)		0.133
Fever						
Days		2.2 ± 1.6 (9)		2.1 ± 1.7 (74)		0.905
Highest temperature, °C		39.0 ± 0.8 (9)		39.4 ± 0.8 (74)		0.236
Urine examinations						
Gravity		1.018 ± 0.009 (22)		1.020 ± 0.009 (139)		0.297
PH		6.2 ± 0.5 (22)		6.3 ± 0.6 (139)		0.567
Protein ^a^		65.0 ± 106.6 (22)		19.8 ± 45.2 (139)		0.256
RBC (/µL)		403 ± 473 (22)		250 ± 367 (139)		0.176
WBC (/µL)		12 ± 7 (22)		10 ± 8 (139)		0.123
Blood						
WBC (/mm^3^)		12,579 ± 5216 (15)		10,669 ± 5372 (74)		0.108
Hb (g/dL)		12.3 ± 1.7 (15)		12.6 ± 1.5 (73)		0.859
PLT (×10^3^/mm^3^)		264.533 ± 89.443 (15)		272.192 ± 82.128 (73)		0.811
Neutrophil (%)		63.4 ± 17.7 (15)		58.9 ± 16.1 (74)		0.278
BUN (mg/dL)		12.1 ± 3.6 (9)		12.4 ± 3.5 (23)		0.849
Creatinine (mg/dL)		0.59 ± 0.18 (12)		0.51 ± 0.25 (47)		0.134
CRP (mg/dL)		3.96 ± 9.91 (14)		2.73 ± 4.03 (71)		0.247
PT(s)		11.0 ± 0.6 (4)		11.2 ± 0.6 (11)		0.947
APTT(s)		31.3 ± 1.0 (4)		28.9 ± 2.9 (11)		0.226
Days of hospitalization		4.6 ± 2.3 (7)		4.8 ± 3.9 (38)		0.830

RBC: red blood cell; OB: occult blood; WBC: white blood cell; Hb: hemoglobin; PLT: platelet; CRP: C-reactive protein; ^a^ *p*-value < 0.05.

**Table 4:** The correlation of variables with different diagnoses in children aged more than or equal to 2 years causing hematuria without pyuria (N = 123).

Variables		UTI (N = 17)		Other Causes, non-UTI (N = 106)		*p*-Value
		N	%	N	%	
Age						
2–7		7	41.2	60	56.6	0.236
7–13		1	5.9	23	21.7	0.231
13–18 ^a^		9	52.9	23	21.7	0.006
Male sex		5	29.4	46	43.4	0.411
Clinical symptoms						
Fever		4	23.5	43	40.6	0.283
Gross hematuria		7	41.2	44	41.5	0.979
Cough		1	5.9	19	17.9	0.371
Rhinorrhea		1	5.9	19	17.9	0.371
Nausea or vomiting		1	5.9	12	11.3	0.801
Diarrhea		0	0	1	0.94	1.000
Urine discharge		0	0	1	100	0.293
Urine frequency		3	17.6	7	6.6	0.285
Dysuria ^a^		11	64.7	28	26.4	0.002
Upper or periumbilical pain		0	0	5	4.7	0.800
Lower abdominal pain		3	17.6	7	6.6	0.285
Flank pain		4	23.5	13	9.4	0.384
Hospitalization		4	23.5	22	20.8	1.000
Urine examinations						
Urine OB	+	14	82.3	66	62.3	0.181
Urine protein	+	12	70.6	56	52.8	0.172
Urine nitrite	+	0	0	2	1.9	1.000
Urine esterase	+	3	17.6	4	3.8	0.084
Urine bacteria	+	0	0	3	2.8	1.000
Cutoff values						
Age> 8 years ^a^		10	58.8	32	30.2	0.021
Urine protein > 30mg/dL ^a^		7	41.2	11	10.4	0.001
Urine RBC > 373/µL ^a^		9	52.9	30	28.3	0.043
Variables’ population means		Mean ± SD (Number)		Mean ± SD (Number)		
Age (years) ^a^		10.4 ± 5.6 (17)		7.2 ± 5.1 (106)		0.030
Fever						
Days		3.0± 1.8 (4)		2.2 ± 1.9 (43)		0.236
Highest temperature, °C		39.0 ± 0.8 (4)		39.4 ± 0.7 (43)		0.552
Urine examinations						
Gravity		1.022 ± 0.007 (17)		1.018 ± 0.009 (106)		0.112
PH		6.3 ± 0.4 (17)		6.4 ± 0.6 (106)		0.484
Protein ^a^		83.5 ± 115.3 (17)		22.4 ± 50.9 (106)		0.045
RBC (/µL)		516 ± 483 (17)		314 ± 425 (106)		0.059
WBC (/µL)		11 ± 6 (17)		9 ± 7 (106)		0.138
Blood						
WBC (/mm^3^)		12,255 ± 3950 (11)		10,533 ± 4321 (54)		0.149
Hb (g/dL)		12.7 ± 1.6 (11)		12.9 ± 1.4(54)		0.739
PLT (×10^3^/mm^3^)		269.000 ± 47.722 (11)		275.463 ± 77.124 (54)		1.000
Neutrophil (%)		67.3 ± 13.0 (11)		62.7 ± 13.9 (54)		0.416
BUN (mg/dL)		12.7 ± 3.5 (7)		12.3 ± 3.5 (23)		0.941
Creatinine (mg/dL)		0.64 ± 0.16 (10)		0.57 ± 0.24 (37)		0.198
CRP (mg/dL)		1.49 ± 2.25 (10)		2.63 ± 4.06 (52)		0.237
PT (s)		11.0 ± 0.7 (3)		11.2 ± 0.6 (11)		0.874
APTT (s)		31.5 ± 1.1 (3)		28.9 ± 2.9 (11)		0.170
Days of hospitalization		3.8 ± 1.5 (4)		5.3 ± 5.0 (22)		0.741

RBC: red blood cell; OB: occult blood; WBC: white blood cell; Hb: hemoglobin; PLT: platelet; CRP: C-reactive protein; ^a^ *p*-value < 0.05.

**Table 5:** The correlation of variables with different diagnoses in children aged less than 2 years causing hematuria without pyuria (N = 38).

Variables		UTI (N = 5)		Other Causes, non-UTI (N = 33)		*p*-Value
		N	%	N	%	
Male sex		4	80	12	36.4	0.175
Clinical symptoms						
Fever		5	100	31	93.4	1.000
Cough		0	0	6	18.2	0.703
Rhinorrhea		0	0	6	18.2	0.703
Nausea or vomiting		1	20	3	9.1	1.000
Diarrhea		0	0	2	6.1	0.583
Gross hematuria		1	20	2	6.1	0.851
Hospitalization		3	60	15	45.5	0.899
Urine examinations						
Urine OB	+	3	60	13	39.4	0.701
Urine protein	+	1	20	17	51.5	0.404
Urine nitrite ^a^	+	2	40	0	0	0.008
Urine esterase ^a^	+	2	40	1	3.0	0.049
Urine bacteria	+	1	20	1	3.0	0.611
Cutoff values						
Urine RBC > 8/uL ^a^		2	40	30	90.9	0.024
Variables’ population means		Mean ± SD (Number)		Mean ± SD (Number)		
Age (years)		0.6 ± 0.5 (5)		0.7 ± 0.5 (33)		0.793
Fever						
Days		1.6 ± 1.3 (5)		1.9 ± 1.2 (31)		0.342
Highest temperature, °C		39.0 ± 0.8 (5)		39.4 ± 0.9 (31)		0.311
Urine examinations						
Gravity		1.014 ± 0.012 (5)		1.018 ± 0.008 (33)		0.385
PH		5.8 ± 0.7 (5)		5.9± 0.5 (33)		0.491
Protein		2.0 ± 4.5 (5)		11.8 ± 14.9 (33)		0.145
RBC (/µL)		20 ± 25 (5)		47 ± 171 (33)		0.123
WBC (/µL)		15 ± 9 (5)		12 ± 8 (33)		0.375
Blood						
WBC (/mm^3^)		13,472.5 ± 8574.4 (4)		11,039 ± 7653 (20)		0.575
Hb (g/dL)		10.9 ± 1.6 (4)		11.8 ± 1.3 (19)		0.239
PLT (×10^3^/mm^3^)		252.250 ± 171.661 (4)		262.895 ± 96.657 (19)		0.903
Creatinine (mg/dL)		52.4 ± 26.1 (4)		48.8 ± 17.7 (20)		0.615
CRP (mg/dL)		10.13 ± 18.41 (4)		3.01 ± 4.04 (19)		0.715
Days of hospitalization		5.7 ± 3.1 (3)		4.1 ± 1.6 (15)		0.427

RBC: red blood cell; OB: occult blood; WBC: white blood cell; Hb: hemoglobin; PLT: platelet; CRP: C-reactive protein; ^a^ *p*-value < 0.05.

## Data Availability

The original contributions presented in this study are included in the article; further inquiries can be directed to the corresponding author.

## Abbreviations

UTI, urinary tract infection; ED, emergency department; RBCs, red blood cells; WBCs, white blood cells; CFUs, colony-forming units; ANA, antinuclear antibody; ASLO, antistreptolysin O; SD, standard deviation; CI, confidence intervals; CRP, C-reactive protein; PCT, procalcitonin.
